# Hypoxia with inflammation and reperfusion alters membrane resistance by dynamically regulating voltage-gated potassium channels in hippocampal CA1 neurons

**DOI:** 10.1186/s13041-021-00857-9

**Published:** 2021-09-23

**Authors:** Yoon-Sil Yang, Joon Ho Choi, Jong-Cheol Rah

**Affiliations:** 1 Korea Brain Research Institute, 61 Cheomdan-ro, Dong-gu, Daegu, 41062 South Korea; 2 Department of Brain & Cognitive Sciences, Daegu Gyeongbuk Institute of Science and Technology, 333 Techno Jungang-daero, Dalseong-gun, Daegu, 42988 South Korea

**Keywords:** Hypoxia, Inflammation, A-type potassium channel, Delayed rectifier potassium channel, Input resistance

## Abstract

Hypoxia typically accompanies acute inflammatory responses in patients and animal models. However, a limited number of studies have examined the effect of hypoxia in combination with inflammation (Hypo-Inf) on neural function. We previously reported that neuronal excitability in hippocampal CA1 neurons decreased during hypoxia and greatly rebounded upon reoxygenation. We attributed this altered excitability mainly to the dynamic regulation of hyperpolarization-activated cyclic nucleotide-gated cation (HCN) channels and input resistance. However, the molecular mechanisms underlying input resistance changes by Hypo-Inf and reperfusion remained unclear. In the present study, we found that a change in the density of the delayed rectifier potassium current (I_DR_) can explain the input resistance variability. Furthermore, voltage-dependent inactivation of A-type potassium (I_A_) channels shifted in the depolarizing direction during Hypo-Inf and reverted to normal upon reperfusion without a significant alteration in the maximum current density. Our results indicate that changes in the input resistance, and consequently excitability, caused by Hypo-Inf and reperfusion are at least partially regulated by the availability and voltage dependence of K_V_ channels. Moreover, these results suggest that selective K_V_ channel modulators can be used as potential neuroprotective drugs to minimize hypoxia- and reperfusion-induced neuronal damage.

**Supplementary Information:**

The online version contains supplementary material available at 10.1186/s13041-021-00857-9.

## Introduction

Hypoxia typically accompanies inflammatory responses in patients with stroke or ischemia and in animal models through hypoxia-inducible factor (HIF) and nuclear factor-kB (NF-kB) [1–6]. Interestingly, the inverse situation also occurs. Various inflammatory responses of the brain cause hypoxia by reducing cerebral blood flow [7]. Therefore, to study the effects of hypoxia on pathophysiological conditions more accurately, the combinatorial effect of hypoxia with inflammation (Hypo-Inf) should be considered. However, only a handful of studies have examined the effects of Hypo-Inf on the regulation of neuronal properties and excitability [8, 9]. In our previous study, we demonstrated a rapid decrease in neuronal excitability during Hypo-Inf and hyperexcitability upon reoxygenation [9]. We suggest that the hyperexcitability observed upon reperfusion can account for postischemic seizures [10–12], and the molecular mechanisms underlying this change in excitability can provide targets for the prevention of reperfusion injury. We attributed the excitability change mainly to hyperpolarization-activated cyclic nucleotide-gated cation (HCN) channels and changes in input resistance. Input resistance was decreased by Hypo-Inf and increased to a higher level than normal upon reperfusion, suggesting that input resistance can be an additional determinant of neuronal excitability. However, the molecular mechanisms underlying input resistance changes remain to be determined. Such input resistance changes are likely independent of the hyperpolarization-activated current (I_h_) change because I_h_ changes in an opposite direction relative to the input resistance. As HCN channels will be partially open constitutively at the resting membrane potential (RMP), the recruitment of HCN channels would decrease the input resistance [13, 14], which is opposite to our observation.

One of the potential candidates for regulating membrane resistance during hypoxia and reperfusion is the voltage-gated potassium (K_V_) channel because K_V_ channels are crucial for maintaining neuronal excitability and are affected by pathological conditions, including stroke and epilepsy [15–18]. In fact, although various studies have suggested the involvement of K_V_ channels, it remains controversial how they are affected in these conditions.

Based on their kinetic properties, K_V_ channels can be categorized into A-type potassium (I_A_) channels, which are transient or rapidly inactivating K^+^ channels, and delayed rectifier potassium (I_DR_) channels, which show slow or non-inactivating currents. I_A_ channels, primarily formed by K_V_4 family channels, regulate input resistance, membrane excitability and synaptic plasticity [19, 20]. In corroboration with the idea that K_V_ channels are responsible for input resistance changes with Hypo-Inf and reperfusion, fewer I_A_ channels and thereby increased excitability upon posthypoxic reperfusion have been reported [21–23]. On the other hand, other researchers have noted the opposite effect [24–26]. To make the problem even more complex, conflicting results have been reported among studies on the I_A_ channel’s role in inflammation [27, 28]. Likewise, the effect of hypoxia and inflammation on I_DR_ channels remains controversial [24, 27, 29–36].

The apparent conflicts regarding the involvement of K_V_ channels might be due to the aforementioned mutual inducibility of hypoxia and inflammation in addition to the diverse time points at which hypoxia and reperfusion have occurred in various specimen preparations across experiments. To resolve these conflicts, we investigated the combined effects of hypoxia and inflammation on K_V_ channels during hypoxia and reoxygenation. We found that the density of I_DR_ channels increased during Hypo-Inf and was reduced to lower than the normal level during reperfusion. Additionally, significantly less inactivation of I_A_ channels was observed at the physiological RMP. These results suggest that, at least in part, the altered input resistance may be due to the dynamic regulation of K_V_ channels in CA1 pyramidal neurons of the hippocampus.

## Materials and methods

### Slice preparation

Acute hippocampal slices were prepared from Sprague-Dawley rats that were between 14 and 21 postnatal days old (n = 10). All animal care and treatment protocols were approved by the Animal Care and Use Committee of the Korea Brain Research Institute (KBRI IACUC no. IACUC-18-00028). Rats were decapitated after euthanasia with CO_2_. The brains were rapidly removed and immersed in an ice-cold cutting solution of the following composition (in mM): choline chloride, 110; KCl, 2.5; NaHCO_3_, 25; NaH_2_PO_4_, 1.25; glucose, 25; CaCl_2_, 0.5; MgCl_2_·6H_2_O, 7; sodium ascorbic acid, 11.6; and pyruvic acid, 3. Thereafter, 300-μm-thick coronal slices were prepared using a vibratome (Leica, Germany). The slices were then incubated for 30 min at 32 °C in artificial cerebrospinal fluid (ACSF) containing the following (in mM): NaCl, 119; KCl, 2.5; NaHCO_3_, 26; NaH_2_PO_4_, 1.25; glucose, 20; CaCl_2_, 2; MgSO_4_, 1; ascorbic acid, 0.4; and pyruvic acid, 2. During sectioning, the solutions were oxygenated with 95% O_2_ and 5% CO_2_.

### Electrophysiological recordings

The slices were transferred to a submerged recording chamber with a continuous flow of ACSF saturated with carbogen (95% O_2_, 5% CO_2_) for whole-cell patch-clamp recordings. Slices were visualized using a BX51 WI microscope (Olympus, Japan) through a 40× water-immersed objective (numerical aperture 0.8). Patch electrodes with 3–5 MΩ tip resistances were prepared using a pipette puller (Sutter Instruments, USA) and filled with internal solution, which was composed of the following (in mM): KCl, 20; potassium gluconate, 125; HEPES, 10; NaCl, 4; EGTA, 0.5; ATP, 4; TrisGTP, 0.3; and phosphocreatine, 10 (pH 7.2, 290–300 mOsm). The liquid junction potential (LJP) between the internal solution and ACSF was 14.523 mV. The data shown in the present study did not take into account the LJP. The cell capacitances were obtained digitally by using Multiclamp software.

Current-clamp recordings were used to measure the input resistance and action potential (AP) firing rate. Input resistance was determined from the slope of the I–V relationship, which was revealed by plotting the amplitude of the steady-state voltage induced by the hyperpolarizing current injection (− 200 pA to − 50 pA, in 50-pA increments), and analyzed using Microsoft Excel. The AP firing rate was obtained by counting the number of APs evoked by 100-pA depolarizing current injection for 600 ms.

To record the currents of K_V_ channels, 0.5 μM tetrodotoxin (TTX) was added to the ACSF to block sodium channels, and voltage-clamp recordings were carried out. The density of potassium channels was obtained by dividing the current by the membrane capacitance. I_A_ was isolated by subtracting the I_DR_ from the total outward potassium current as follows. The total outward currents were elicited by a depolarizing pulse to + 60 mV for 400 ms, following a holding potential at − 60 mV. The I_DR_ was measured by applying a depolarizing pulse to + 60 mV for 400 ms after a − 20 mV prepulse for 200 ms to eliminate I_A_. To measure the density of I_DR_, the membrane potentials were held at − 20 mV for 200 ms to remove I_A_ and then depolarized to + 80 mV for 400 ms. To determine the inactivation kinetics of I_A_, voltage-dependent inactivation was assessed by measuring the peak amplitude of current responses by depolarizing to + 60 mV after the prepulse to potentials between − 100 and − 20 mV in 10-mV increments. The normalized current was fitted by the Boltzmann equation:

$${\text{I}}/{\text{I}}_{{{\text{max}}}} = {1}/\left\{ {{1} + {\text{exp}}\left[ {\left( {{\text{V}}_{{\text{m}}} - {\text{V}}_{{\text{h}}} } \right)/{\text{k}}} \right]} \right\},$$

where V_h_ is the half-maximal membrane potential for inactivation and k is the slope factor. The activation kinetics of the I_DR_ were studied by depolarizing pulses (− 60 mV to + 80 mV in 20-mV increments) following a − 20 mV prepulse to eliminate I_A_. The I_DR_ was converted into conductance using the formula

$${\text{G}} = {\text{I}}/\left( {{\text{V}}_{{\text{m}}} - {\text{V}}_{{{\text{rev}}}} } \right).$$

The conductance of I_DR_ was normalized and then fitted by a Boltzmann equation.

All electrophysiological recordings were performed at 32 °C, lowpass filtered and digitized at 10 kHz using an Axopatch 700B amplifier (Molecular Devices, Foster City, CA) and Digidata 1550A (Molecular Devices, Foster City, CA).

### Hypoxia with inflammation

Hypoxia was induced by perfusion with ACSF saturated with 8% O_2_, 5% CO_2_, and 87% N_2,_ and 10 μg/mL lipopolysaccharide (LPS) was added to ACSF to induce inflammation. Hypo-Inf was induced by perfusion with 10 μg/mL LPS-containing ACSF saturated with 8% O_2_, 5% CO_2_, and 87% N_2_. To examine the effect of reoxygenation or wash out of the inflammation, the perfusion solution was changed to normally oxygenated ACSF (95% O_2_ and 5% CO_2_) without LPS.

### Data analysis and statistics

Data acquisition and analysis were performed by using Clampfit 10.4 (Axon Instruments, Foster City, CA) and IGOR Pro software (Wavemetrics, Lake Oswego, OR). MS Excel (Microsoft, Redmond, WA) software and GraphPad Prism 7.0 (GraphPad Software, San Diego, CA) were used for further data analysis and statistical analysis. All data are presented as the mean ± standard error (SEM). Statistical significance for comparisons between variables for the same set of neurons, such as control, Hypo-Inf, and reperfusion, was examined using the paired t-test. Otherwise, an unpaired t-test was used. Statistical significance was accepted for p values of < 0.05 or 0.01, as indicated.

## Results

### Changes in input resistance

**Fig. 1:**
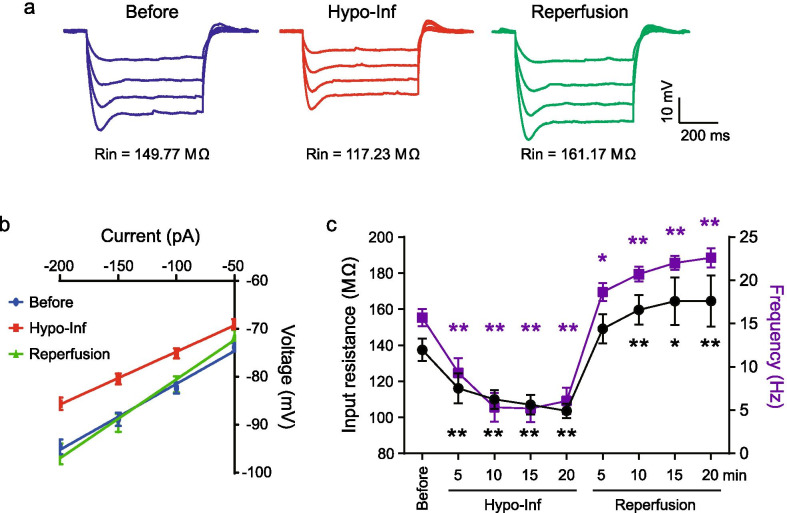
Effects of Hypo-Inf and reperfusion on input resistance of hippocampal CA1 neurons. **a** Sample traces of voltage responses to negative current pulses from − 200 to – 50 pA in 50-pA increments. Scale bars: 10 mV, 200 ms. **b** I–V relationship during Hypo-Inf and reperfusion. **c** Input resistance (black, left axis) and AP frequency (purple, right axis) as a function of time during Hypo-Inf and reperfusion. Note that the AP frequency data were reused from our previous study [9]. Error bars represent standard errors. Significance, relative to before, was determined using paired t-tests (*p < 0.05, **p < 0.01)

We first tested the input resistance of neurons altered by Hypo-Inf and reperfusion in the presence of TTX. Corroborating our previous study [9], we found that the input resistances were significantly decreased by Hypo-Inf (before: 137.46 ± 6.18 MΩ; 15 min after Hypo-Inf: 107.03 ± 5.51, p < 0.01), whereas reperfusion significantly increased the input resistance (15 min after reperfusion: 164.45 ± 13.10 MΩ, p < 0.01; Fig. 1a–c). Of note, the input resistance change coincided with the frequency of APs in response to Hypo-Inf and reoxygenation, suggesting that the input resistance change could contribute to the excitability of the neuron and that voltage-dependent sodium channels were not responsible for this change (Fig. 1c).

### Changes in I_A_ channels

To examine whether I_A_ channels might participate in changes in the input resistance by Hypo-Inf and/or reoxygenation, we first measured the maximum current density of the I_A_ channel. We found that the maximum I_A_ density was unchanged during Hypo-Inf and reperfusion (before: 61.6 ± 5.1 pA/pF; Hypo-Inf: 66.0 ± 6.46 pA/pF; reperfusion: 56.96 ± 6.88 pA/pF; Additional file 1: Figure S1), indicating that input resistance during and after Hypo-Inf did not originate from the availability of I_A_ channels on the surface of the neurons.

**Fig. 2:**
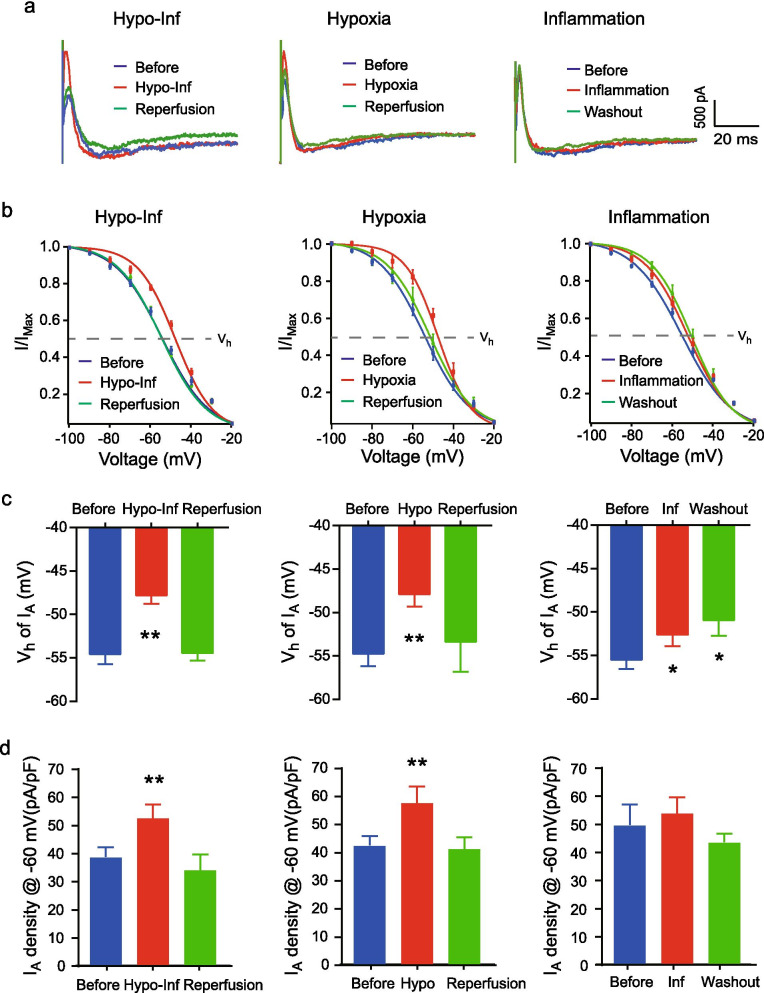
Changes in I_A_ channels with hypoxia with or without inflammation. **a** Example traces of I_A_. Before (blue), insults (red), and reperfusion/washout (green). Scale bars: 500 pA, 20 ms. **b** Boltzmann fitted I_A_ inactivation curves. **c** Averaged voltage half (V_h_, the dotted line in **b**); Left: Hypo-Inf; middle: hypoxia; right: inflammation. **d** The density of I_A_ was measured by depolarization to + 60 mV after a − 60 mV prepulse potential. Error bars represent standard errors. Significance was determined using paired t-tests (*p < 0.05 and **p < 0.01, compared with each before period)

However, the available I_A_ density near the RMP can vary depending on the inactivation kinetics of the channel. To determine the inactivation kinetics of I_A_ channels, I_A_ was elicited by depolarization to + 60 mV after prepulse potentials (− 100 mV to − 20 mV at 10-mV increments). The measured peak currents were normalized with respect to the maximum I_A_ (I/I_max_) and plotted as a function of the prepulse potential. The normalized current–voltage relationship was fitted by the Boltzmann equation (Fig. 2b). We found that the inactivation curves of I_A_ were shifted toward the depolarizing potential by Hypo-Inf without changing the maximum I_A_ density and were returned to the control level by reperfusion (Fig. 2b, left). The voltage at half of the maximum I_A_ (voltage half or V_h_) was significantly increased from − 54.58 ± 1.13 mV before to − 48.23 ± 1.10 mV at 10 min after Hypo-Inf (p < 0.01; Fig. 2c). During reperfusion following Hypo-Inf, the V_h_ became comparable to that before (− 54.44 ± 0.85 mV, p = 0.4851). As a result, the I_A_ density near the RMPs (− 61.51 ± 0.97 mV) was enhanced during Hypo-Inf (before: 38.88 ± 3.27 pA/pF; Hypo-Inf: 47.83 ± 4.31 pA/pF, p < 0.01) but not during reperfusion (35.28 ± 3.68 pA/pF, Fig. 2d, left). These results suggested that I_A_ channels were involved in downregulating neuronal excitability by shifting the inactivation curve of I_A_ channels during Hypo-Inf.

To determine whether the shifted inactivation can be solely explained by either hypoxia or acute inflammatory responses, we also measured the inactivation of I_A_ channels under the two separate insults (Fig. 2, middle column). During hypoxia, the inactivation curves of I_A_ were shifted in a similar manner as those during Hypo-Inf (V_h_: − 54.73 ± 1.43 mV before and − 47.08 ± 1.58 mV at 10 min after the hypoxia insult) and returned to the control level during reperfusion (− 53.36 ± 3.47 mV). Therefore, the I_A_ density near RMPs was altered by hypoxia alone (before hypoxia: 42.67 ± 3.08 pA/pF; hypoxia: 57.79 ± 5.43, p < 0.01).

Unexpectedly, the inactivation curves of I_A_ slightly but significantly shifted toward a depolarizing potential during both the inflammation and washout afterward (Fig. 2, right column; V_h_ before: − 55.50 ± 1.05 mV; V_h_ inflammation: − 52.62 ± 1.34 mV, p < 0.01) and stayed depolarized following washout (V_h_ − 49.94 ± 1.82 mV, p < 0.01). However, the I_A_ density near RMP was unchanged by LPS alone.

These results indicated that the I_A_ density near RMP was involved in the reduced excitability upon Hypo-Inf, not by changes in the density but by the shifted inactivation kinetics of the channels.

### Density of the I_DR_ channels

**Fig. 3:**
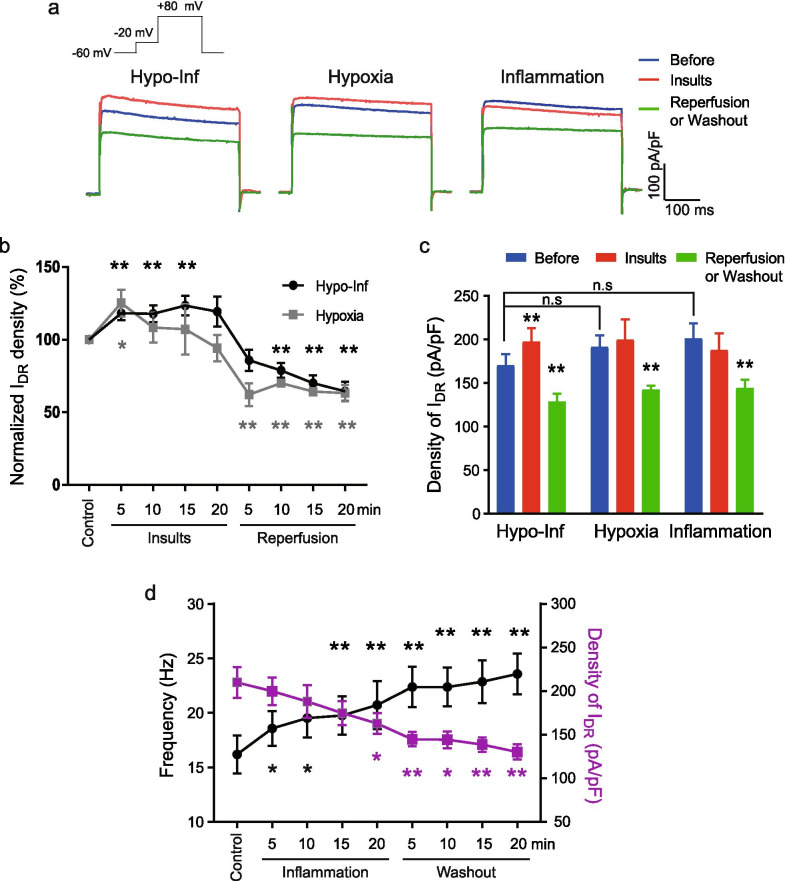
Changes in I_DR_ density by Hypo-Inf and reperfusion. **a** Example traces of I_DR_ measured before (control, blue), during (insults, red), and with reperfusion or washout (green); Left: Hypo-Inf; middle: hypoxia; right: inflammation. Scale bars: 100 pA/pF, 50 ms. **b** The normalized I_DR_ density with Hypo-Inf (black) or hypoxia alone (gray) and reperfusion. **c** Comparison of the density of I_DR_ following different insults: before (blue), insults (red), and reperfusion (green). **d** The change in firing frequency in response to 100-pA current injection (left axis and black circles) and I_DR_ density (right axis and gray squares) with inflammation and washout (*p < 0.05 and **p < 0.01, compared with each before period)

**Fig. 4:**
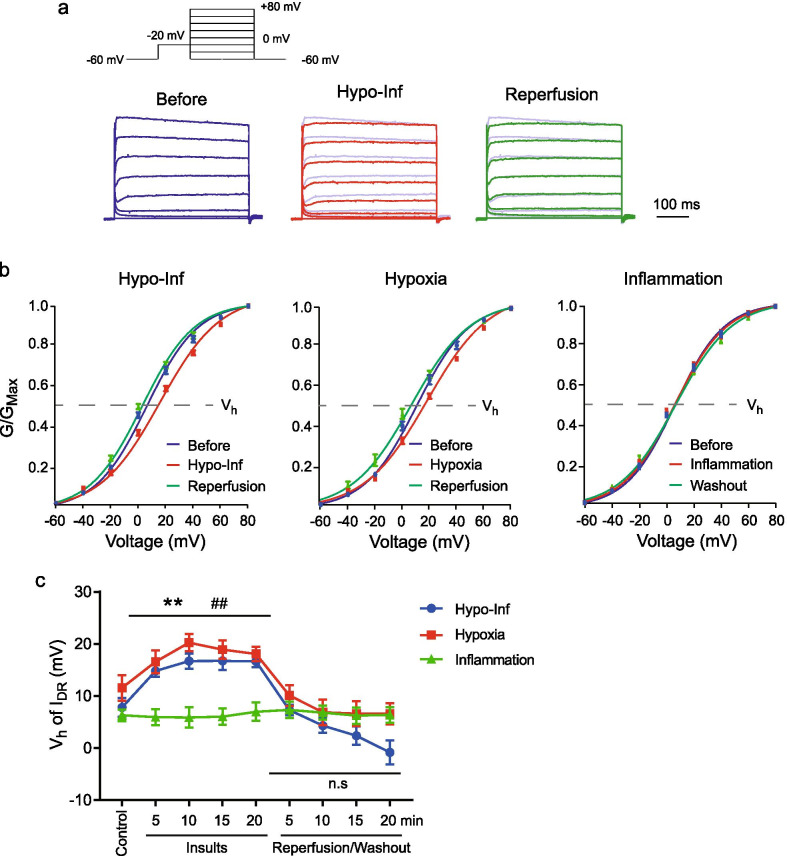
Activation kinetics of I_DR_ with Hypo-Inf, hypoxia, and inflammation. **a** Example traces of normalized I_DR_. Each trace shows control (blue), Hypo-Inf (red), and reperfusion (green). Scale bars: 100 ms. **b** Fitted Boltzmann curves of gating kinetics of I_DR_ activation; Left: Hypo-Inf; middle: hypoxia; right: inflammation. **c** Averaged values of voltage half (V_h_, the dotted line in **b**) over the course of control, insults, and reperfusion. Error bars represent standard errors. Significance was determined using paired t-tests (**p < 0.01 and ^##^p < 0.01, compared with before Hypo-Inf and hypoxia, respectively)

We next examined the density of I_DR_, the steady-state current of I_DR_ normalized by whole-cell capacitance, by hypoxic and/or inflammatory insults. The I_DR_ density changed to account for the alteration in input resistance and, thereby, the excitability (Fig. 4). In agreement with the idea that the input resistance was influenced by the I_DR_ density, the I_DR_ density was significantly enhanced during Hypo-Inf (Fig. 3a–c; before: 169.72 ± 11.55 pA/pF; 15 min after Hypo-Inf: 197.59 ± 14.18 pA/pF, p < 0.01) and was significantly reduced during reperfusion after Hypo-Inf (15 min after reperfusion: 119.90 ± 12.80 pA/pF, p < 0.01).

We previously demonstrated that excitability changes induced during Hypo-Inf and reperfusion were closely replicated by hypoxia alone [9]. Thus, we also examined whether the changes in I_DR_ density may be explained by hypoxia alone. Indeed, an increase in I_DR_ density was observed with hypoxia (before: 191.86 ± 11.08 pA/pF; 5 min after hypoxia: 240.75 ± 21.10 pA/pF, p < 0.1). However, the increased I_DR_ density with hypoxia alone was not sustained as long as that with Hypo-Inf (Fig. 3b). The I_DR_ density measured at 10, 15, and 20 min after hypoxia without inflammation was similar to that of the control (200.18 ± 19.43, 189.38 ± 13.01, and 170.68 ± 13.97 pA/pF; p = 0.49, 0.88, and 0.42, respectively). These results suggested that hypoxia was responsible for the initial increase in I_DR_ density, but the combinatorial effect of LPS on proinflammatory responses unexpectedly extended the effects of hypoxia on I_DR_ density.

Decreased I_DR_ density by reperfusion after Hypo-Inf could be replicated by reperfusion following hypoxia alone (15 min after reperfusion: 132.04 ± 6.70 pA/pF, p < 0.01). In our previous study, we observed that excitability was irreversibly increased by inflammation [9]. Therefore, here, we examined whether I_DR_ density changes could explain this phenomenon. The I_DR_ density was slowly reduced and became significant after 20 min (before: 201.26 ± 15.12 pA/pF; 20 min after inflammation: 162.84 ± 10.69 pA/pF, p < 0.05; Fig. 3d) and remained reduced after washout (138.89 ± 7.39, p < 0.01). These results suggested that a decrease in I_DR_ could be one of the sources of hyperexcitability caused by inflammation.

### Changes in the activation kinetics of I_DR_

The steady-state activation kinetics of I_DR_ channels were determined by depolarizing pulses between − 60 and + 80 mV in 20-mV increments for 400 ms, following a − 20 mV prepulse for 200 ms to remove I_A_ (Fig. 4a). The conductance was normalized to the maximum value and then fitted with the Boltzmann equation. Hypo-Inf shifted the activation curves of I_DR_ in the depolarizing direction (V_h_: 7.81 ± 1.83 mV before Hypo-Inf and 16.76 ± 1.78 mV at 15 min after Hypo-Inf, p < 0.01; Fig. 4c) and returned to the control level with reperfusion (V_h_: 2.39 ± 1.78 mV, p = 0.2801 at 15 min, − 0.813 ± 2.28 mV, p = 0.069 at 20 min). The activation of I_DR_ channels was similarly changed by hypoxia alone and reperfusion (V_h_: 11.57 ± 2.45 mV before hypoxia and 18.90 ± 1.77 mV at 15 min after hypoxia, p < 0.01; V_h_: 6.62 ± 2.37 mV with reperfusion, p = 0.2324, compared with control). The activation kinetics of I_DR_ channels were not affected by inflammation (V_h_ before inflammation: 6.30 ± 1.10 mV; 15 min after inflammation: 6.06 ± 1.54 mV; 15 min after reperfusion: 6.25 ± 1.58 mV). These results suggested that the increased I_DR_ density could not be attributed to the activation kinetics of the channel, which was different from previous observations [34, 35].

## Discussion

In the current study, we explored the contribution of K_V_ channels to the previously observed alterations in input resistance and neuronal excitability by combined insults to hypoxia and inflammation and reperfusion. In line with our previous study, we focused our analysis on the effect of hypoxia and inflammation on hippocampal pyramidal neurons. The hippocampus is one of the brain areas most vulnerable to acute ischemic damage. This vulnerability is perhaps due to high energy consumption and restricted blood oxygen saturation [37–40]. Furthermore, ischemic damage in the hippocampus often leads to long-lasting problems, including memory loss and vascular dementia [41–44].

Reportedly, the experimental conditions closely mimic ischemia, including the depletion of ATP evidenced by immediate adenosine receptor-dependent decreases in neurotransmitter release efficiency [9, 45, 46] and activation of microglial complement receptor 3 (CR3) [8]. CR3 activation in turn produces reactive oxygen species by activating NADPH oxidase, which activates a protein phosphatase critical for long-term synaptic depression [47, 48]. Under these conditions, excitability changes accompanied by I_h_ current and input resistance were observed [9].

The input resistance of neurons reflects the ability of ions to penetrate the membrane and mainly relies on the gating of ion channels, including HCN, Na_V_, and K_V_ channels [13, 14, 49, 50]. We first excluded the HCN channel because I_h_ changed in the opposite way to explain the input resistance alterations; that is, there was increased I_h_ with increased input resistance and decreased I_h_ with decreased input resistance [9]. Furthermore, the I_h_ channel blocker zatebradine did not reverse the input resistance change by Hypo-Inf or reperfusion [9]. Na_V_ is unlikely to be associated with input resistance because the input resistance was changed by Hypo-Inf and reperfusion in the same manner in the presence of TTX (Fig. 1). Therefore, in the present study, we focused on the role of K_V_ channels in determining the input resistance during Hypo-Inf and reperfusion. K_V_ channels are a prime candidate because the current density and gating kinetics are dynamically regulated by phosphorylation states [51–54], which are altered in response to ischemia [55–57].

In particular, the contribution of I_A_ channels to excitability in hypoxia and reperfusion has attracted attention because of the dynamic regulation of their surface expression and dominant roles in determining excitability [19, 20]. Indeed, numerous studies have suggested the direct involvement of K_V_ channels in ischemia-induced activity changes but with no clear agreement [21–26, 29–36]. Many of these conflicts could be attributed to the differential time points of the assessments during the course of hypoxia and reperfusion based on various methods of specimen preparation [24–29, 32–39]. The effects of hypoxia on K_V_ channels were assessed by measuring the properties of I_A_ and I_DR_ many hours after posthypoxic reperfusion in some studies [21–26], whereas others examined them immediately after or during the hypoxic insult [29–31, 34, 35]. Potassium currents were measured from cultured neurons under chemical ATP depletion in some studies, while in other studies, the currents were measured from brain slices or in vivo with an insufficient oxygen supply. Furthermore, the concurrent effect of inflammation has been mostly ignored in the majority of in vitro studies [22, 29, 34, 35, 54]. To resolve this disagreement, we continuously monitored the changes in K_V_ channel properties throughout hypoxia and reperfusion. Furthermore, we examined the combined (Hypo-Inf) and independent (hypoxia or inflammation) effects of hypoxia and inflammation and reperfusion thereafter.

We found a significantly greater availability of I_A_ channels during Hypo-Inf near the RMPs (Fig. 2) without alterations in the maximum I_A_ density. The increased availability of I_A_ was attributed to the less voltage-sensitive inactivation of I_A_ channels during Hypo-Inf (Fig. 2b, c). Additionally, we found that the observed changes in I_A_ channels by Hypo-Inf were caused by hypoxia.

To examine whether the absence of a change in the maximum I_A_ density in Hypo-Inf could be explained by a combinatorial effect with inflammation, we examined I_A_ with hypoxia or inflammation separately. However, no significant changes in I_A_ density were found under conditions of hypoxia or inflammation alone. It must be noted that our analysis was focused on the acute effect of hypoxia or hypoxia with LPS-induced inflammation. The longer-term impact of inflammation, especially hypoxia-derived endogenous inflammation in vivo*,* should be examined in future studies.

We then tested whether changes in I_DR_ density were involved in the altered input resistance produced by Hypo-Inf and reperfusion. Our results demonstrated that the I_DR_ density changed in a manner that explained the input resistance changes with Hypo-Inf and reperfusion (Fig. 3). We could not identify a causal relationship between I_DR_ and input resistance in the current study due to the lack of a method to selectively inhibit I_DR_ density changes with Hypo-Inf. However, previous studies using a genetic mutation of K_v_2 channels, a major component of the I_DR_ in hippocampal pyramidal neurons, have proven the role of I_DR_ channels in determining input resistance [58, 59].

Interestingly, the effect of Hypo-Inf was not a simple sum of the two individual insults, hypoxia and inflammation. I_DR_ density was initially increased by hypoxia alone but returned to the control levels in ~ 10 min, while inflammation alone had no direct effect on I_DR_ density. The extended effect of inflammation may have been due to the mutually inducible interaction between hypoxia and inflammation. The complexity of the combined response by Hypo-Inf may be inherited from the complexity of reactive oxygen species (ROS)-mediated excitability regulation. Inflammation and hypoxia, as well as reperfusion, can induce the generation of ROS [60–62]. Enhanced excitatory synaptic transmission and excitability in various pathological conditions and pain transduction have been reported [63, 64]. In contrast, increased IDR reduced excitability and inhibited voltage-gated Ca2+ channels were observed in cardiac neurons [65]. Long-term synaptic depression by internalization of glutamate receptors has been reported to mediate ROS signaling during Hypo-Inf [8]. The exact mechanism underlying the elongation of I_DR_ density changes by inflammation under our experimental conditions needs to be further explored in future studies.

On the other hand, reperfusion-induced hyperexcitability correlated with a marked reduction in I_DR_ density. The downregulation of I_DR_ channels depolarizes the RMP and increases the input resistance, as observed in our previous study [9]. Our results were consistent with previous findings of posthypoxic reperfusion-induced epileptic neural activities in vitro and in vivo [21, 22, 66]. Supporting the idea that hyperexcitability can be driven by the regulation of I_DR_ channels, animal models lacking K_V_2.1 show enhanced susceptibility to epileptic neural activity and hyperactive behavior [67]. Moreover, mutations in the K_V_2.1 channel have been reported as genetic causes of epileptic encephalopathy in human patients [59, 68, 69]. Therefore, we concluded that the dynamic regulation of I_DR_ density regulates the excitability of neurons during hypoxia and reperfusion.

The detailed molecular mechanism underlying the regulation of I_DR_ density is beyond the scope of the current study, but the conductance control of I_DR_ through distribution patterns of the channels has recently attracted attention. It has been observed that dispersed K_V_2.1 channels conduct potassium ions more efficiently than clustered K_V_2.1 channels [70, 71]. Clustering of the K_V_2.1 channel is regulated through activity-dependent phosphorylation by cyclin-dependent kinase 5 (Cdk5) [72]. Indeed, enhanced activity of Cdk5 has been reported after hypoxia/ischemic injury [73, 74].

We next tested whether changes in I_DR_ densities by Hypo-Inf and reperfusion were due to the altered voltage dependency of the channel activation. Previous studies observed that ischemia dephosphorylated K_V_2.1 channels through calcineurin and declustered them, consequently shifting the voltage-dependent activation of I_DR_ channels toward the hyperpolarizing direction [34, 35]. However, we observed that Hypo-Inf, as well as hypoxia alone, induced a depolarizing shift regarding the activation of I_DR_ channels, despite the enhanced I_DR_ density (Fig. 4). The shifted activation curves of I_DR_ by Hypo-Inf could be a result of compensation for the rapidly increased I_DR_ density. This discrepancy can be explained by experimental differences. Previous studies examined I_DR_ kinetic changes via chemical ATP depletion from cultured neurons and cortical slices [34, 35], whereas we assessed the effect of insufficient oxygen supply in hippocampal neurons in acutely prepared brain slices. Increased I_DR_ density, despite the shifted activation during hypoxia, suggested that the number of I_DR_ channels on the surface may have been actively regulated by the level of oxygen [15, 75, 76].

In conclusion, we demonstrated that excitability altered by hypoxia and reperfusion can be attributed to input resistance changes through dynamic regulation of K_V_ channels. Further work should examine changes in excitability in various brain regions and the dependence of these channels at the system level. Such research would directly evaluate the selective regulation of neuronal K_V_ channels as a therapeutic or neuroprotective method to ameliorate the effects of hypoxia and reperfusion.

## Supplementary Information

**Additional file 1: Figure S1.** The maximum density of IA before, during, and after Hypo-Inf. Error bars represent standard errors.

## Data Availability

The data used in our study are available from the authors upon reasonable request.

## Abbreviations

AC: Adenylyl cyclase
ACSF: Artificial cerebrospinal fluid
AP: Action potential
ATP: Adenosine triphosphate
cAMP: Cyclic adenosine monophosphate
Cdk5: Cyclin-dependent kinase 5
CR3: Complement receptor 3
HCN: Hyperpolarization-activated cyclic nucleotide-gated cation
HIF: Hypoxia-inducible factor
Hypo-Inf: Hypoxia with inflammation
I_A_: A-type potassium current
I_DR_: Delayed rectifier potassium current
I_h_: Hyperpolarization-activated current
K_V_ channel: Voltage-gated potassium channel
LJP: Liquid junction potential
LPS: Lipopolysaccharide
Na_V_ channel: Voltage-gated sodium channel
NF-kB: Nuclear factor-kB
PKA: Protein kinase A
RMP: Resting membrane potential
ROS: Reactive oxygen species
SNARE: Soluble *N*-ethylmaleimide-sensitive factor attachment protein receptors
TLR4: Toll-like receptor 4
TTX: Tetrodotoxin
V_h_: Voltage half
