# Supplementary figures and images for: Hypoxia with inflammation and reperfusion alters membrane resistance by dynamically regulating voltage-gated potassium channels in hippocampal CA1 neurons
Source: Mol Brain. 2021 Sep 23;14:147. doi: 10.1186/s13041-021-00857-9 (PMC8461870; doi:10.1186/s13041-021-00857-9)

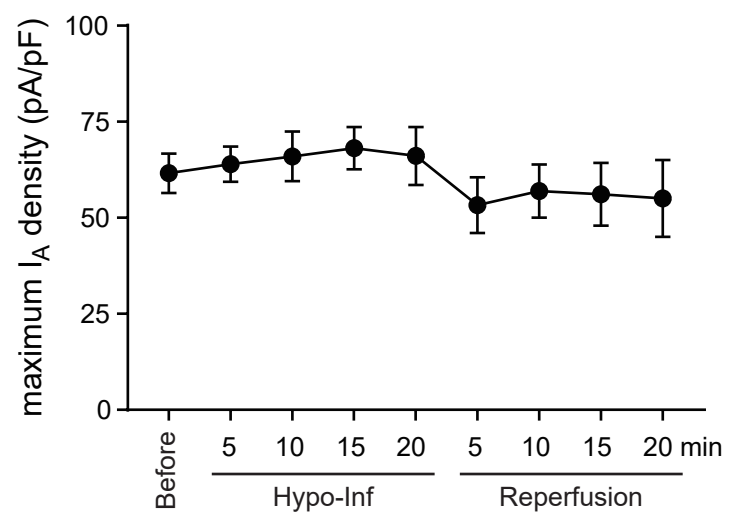

Supplement: Supplementary file 1 — Additional file 1: Figure S1. The maximum density of IA before, during, and after Hypo-Inf. Error bars represent standard errors. [file 13041_2021_857_MOESM1_ESM.pdf]
